# Repeated Administration of Guar Gum Hydrogel Containing Sesamol-Loaded Nanocapsules Reduced Skin Inflammation in Mice in an Irritant Contact Dermatitis Model

**DOI:** 10.3390/pharmaceutics17081029

**Published:** 2025-08-07

**Authors:** Vinicius Costa Prado, Bruna Rafaela Fretag de Carvalho, Kauani Moenke, Amanda Maccangnan Zamberlan, Samuel Felipe Atuati, Ana Clara Perazzio Assis, Evelyne da Silva Brum, Raul Edison Luna Lazo, Andréa Inês Horn Adams, Luana Mota Ferreira, Sara Marchesan Oliveira, Letícia Cruz

**Affiliations:** 1 Laboratório de Tecnologia Farmacêutica, Programa de Pós-Graduação em Ciências Farmacêuticas, Centro de Ciências da Saúde, Universidade Federal de Santa Maria, Santa Maria CEP 97105-900, RS, Brazil; vini132007@gmail.com (V.C.P.); kauanik@hotmail.com (K.M.); 2 Laboratório de Avaliação Biofarmacêutica e Controle de Qualidade, Programa de Pós-Graduação em Ciências Farmacêuticas, Centro de Ciências da Saúde, Universidade Federal de Santa Maria, Santa Maria CEP 97105-900, RS, Brazil; brunarafaela.carvalho@gmail.com (B.R.F.d.C.); amanda.zamberlan@gmail.com (A.M.Z.); andrea.ih.adams@gmail.com (A.I.H.A.); 3 Laboratório de Neurobiologia da Dor, Programa de Pós-Graduação em Ciências Biológicas: Bioquímica Toxicológica, Centro de Ciências Naturais e Exatas, Universidade Federal de Santa Maria, Santa Maria CEP 97105-900, RS, Brazil; atuatis@gmail.com (S.F.A.); ana.perazzio@acad.ufsm.br (A.C.P.A.); evelyne.brum@ufrgs.br (E.d.S.B.); saramarchesan@ufsm.br (S.M.O.); 4 Graduate Program in Biological Sciences: Biochemistry, Department of Biochemistry, Institute of Basic Health Sciences, Universidade Federal do Rio Grande do Sul, Porto Alegre CEP 90035-003, RS, Brazil; 5 Centro de Estudos em Biofarmácia, Programa de Pós-Graduação em Ciências Farmacêuticas, Universidade Federal do Paraná, Curitiba CEP 80210-170, PR, Brazil; raulunalazo@gmail.com (R.E.L.L.); luanamota@ufpr.br (L.M.F.)

**Keywords:** natural gums, parabens, chemometrics analysis

## Abstract

**Background/Objectives**: Dermatitis is frequently treated with dexamethasone cutaneous application, which causes adverse effects mainly when it is chronically administered. Sesamol is a phytochemical compound known for its anti-inflammatory activity and low toxicity. Therefore, this study reports the optimization of a guar gum hydrogel with enhanced physicochemical and microbiological stability, providing an effective dosage form for topical application of sesamol nanocapsules to treat irritant contact dermatitis. **Methods**: Nano-based hydrogel containing 1 mg/g sesamol was prepared by adding the nanocapsule suspension to form a 2.5% (*w*/*v*) guar gum dispersion. Dynamic rheological analysis indicates that the formulations exhibit a non-Newtonian flow with pseudoplastic behavior. Hydrogels were evaluated by Fourier-transformed infrared (FTIR) spectroscopy, and, following spectrum acquisition, an unsupervised chemometrics model was developed to identify crucial variables. Additionally, the physicochemical and microbiological stability of the hydrogel was evaluated over a 60-day period. **Results**: ATR-FTIR spectra of all hydrogels evaluated are very similar after preparation and 60 days of storage. However, it showed a slight increase in average diameter and PDI and decreased pH values after 60 days. Microbiological assessment demonstrated that the hydrogel met the requirements for the microbial count over 60 days. The dermatitis model was induced by repeated applications of croton oil in the right ears of mice. The effectiveness of the hydrogels was evaluated by assessing ear edema and migration of polymorphonuclear cells. The nano-based hydrogel exhibited anti-inflammatory properties similar to those of dexamethasone. **Conclusions**: Therefore, the nano-based hydrogel containing sesamol exhibits therapeutic potential for treating cutaneous inflammatory diseases.

## 1. Introduction

Irritant contact dermatitis is characterized by pruritic lesions, inflammation, edema, redness, excoriation, papules, and subcutaneous nodules [1]. This multifaceted and complex pathology is related to the impairment of the epidermal barrier, which can lead to the penetration of environmental agents and allergens, as well as an imbalance in skin homeostasis [2,3]. The primary therapy chosen in the clinic consists of cutaneous corticosteroid application, either alone or in combination with other pharmacological approaches [4,5]. Although widely used, topical corticosteroids can cause side effects such as stretch marks, adrenal suppression, and increased glucose levels [6,7,8], reinforcing the need to develop new therapeutic approaches.

Hydrogels have gained significant attention as promising topical treatments for dermatitis due to their excellent hydration properties, biocompatibility, and capacity for controlled drug release. Recent studies have demonstrated that polysaccharide-based and stimuli-responsive hydrogels can effectively modulate inflammation, enhance skin repair, and target microbial infections associated with dermatitis [9]. Moreover, innovative hydrogels containing natural bioactive compounds with anti-inflammatory and antibacterial activities have shown clinical efficacy in reducing symptoms and improving patient quality of life [10]. These findings highlight the potential of hydrogels as therapeutic platforms for the therapeutic management of dermatitis.

Phenolic agents have been evaluated as alternatives to existing anti-inflammatory treatments [11,12]. In this context, sesamol had several pharmacological actions, including modulation of oxidative status and anti-inflammatory activity in preclinical studies [13,14]. Considering the promising pharmacological properties reported for sesamol, developing hydrogel designed for its cutaneous application is attractive. Additionally, pharmaceutical dosage forms administered through the cutaneous route offer several advantages compared with other routes, including convenience, increased adherence, reduced systemic adverse effects, and improved effectiveness for drugs with low oral bioavailability [15,16]. Although sesamol exhibits promising pharmacological properties for the treatment of dermatitis, its physicochemical characteristics, such as a low molecular weight (138.1 g/mol) and water solubility (38.8 mg/mL), lead to rapid permeation through the skin layers. As a result, its bioavailability in the skin is limited, compromising its therapeutic efficacy at the injured site [17,18,19].

The nanocapsules, a nanoreservoir particle in which a polymeric shell surrounds an oily core, can constitute a promising alternative for the cutaneous administration of sesamol, improving its pharmacological action and modulating its skin permeation profile at the dermatitis-injured site [20,21]. However, since polymeric nanocapsules are obtained as a liquid suspension, their direct application to affected skin areas can be challenging [22]. To overcome this, a guar gum-based hydrogel incorporating the sesamol-loaded nanocapsules was developed to facilitate cutaneous administration and enhance the practicality of use.

In this context, a previous study by our research group developed a nano-based guar gum hydrogel containing sesamol and evaluated its pharmacological performance in an acute dermatitis model induced by the single application of croton oil [23]. The results demonstrated that the nano-based hydrogel exhibited a therapeutic efficacy similar to that of the reference drug, dexamethasone. Importantly, the free sesamol hydrogel did not present a significant effect, reinforcing the benefit of sesamol nanoencapsulation [23]. The superior effect of the hydrogel containing sesamol nanocapsules can be attributed to the fact that nanoencapsulation increases the residence time of sesamol in the skin layers, as indicated by a permeation profile evaluation, thereby enhancing the pharmacological action observed in the acute dermatitis in vivo model.

Despite the promising pre-clinical evidence in our preceding study, the developed hydrogel presented issues related to its microbiological stability. A few days after its preparation, microbial growth was observed. This was attributed to guar gum, a polysaccharide composed of glucose molecules, which serves as a substrate for microbial growth [24,25].

To optimize the formulation for ongoing studies, this research focused on developing a hydrogel with a preservative system and analyzing its rheological properties and component interactions. Furthermore, building on the promising results observed in the acute model [23], we propose repeated hydrogel administration to further evaluate the benefits of nanoencapsulation in enhancing sesamol’s cutaneous anti-inflammatory effects.

## 2. Materials and Methods

### 2.1. Materials

Sigma Aldrich (St. Louis, MO, USA) commercially supplied the sesamol, sorbitan monooleate (Span^®^ 80), and croton oil. Metachem (São Paulo, SP, Brazil) and Colorcon (Cotia, SP, Brazil) donated the guar gum and ethylcellulose polymer (Ethocel^®^TM Standard 10 Premium Ethylcellulose), respectively. Delaware (Porto Alegre, RS, Brazil) supplied the polysorbate 80 (Tween^®^ 80) and medium chain triglycerides (MCT). Dexamethasone acetate (1 mg/g—Teuto^®^) and methylparaben propylparaben were purchased in a commercial drugstore (Santa Maria, RS, Brazil). Acetone was acquired from CRQ (São Paulo, SP, Brazil). Merck (Darmstadt, Germany) was the supplier of hematoxylin-eosin and paraffin. The high-performance liquid chromatography grade methanol was acquired from LiChrosolv (São Paulo, SP, Brazil). All other reagents and solvents were of analytical grade.

### 2.2. Preparation of Hydrogels

Initially, the nanocapsule suspensions contained sesamol (1 mg/mL) were produced by the interfacial deposition of the preformed polymer method according to our previous study [23]. The organic phase, consisting of ethylcellulose (0.100 g), sorbitan monooleate (Span^®^ 80, 0.077 g), medium chain triglyceride (MCT) oil (0.300 g), and acetone (27 mL), was subjected to magnetic stirring at 40 °C for 1 h. Subsequently, sesamol (0.010 g) was incorporated into the organic phase under continuous stirring. This organic mixture was then slowly introduced into an aqueous solution (53 mL) containing polysorbate 80 (Tween^®^ 80, 0.077 g) while maintaining constant stirring. After 10 min, the acetone was removed, and the resulting suspension was concentrated to a final volume of 10 mL using a rotary evaporator (Rotary evaporator Model 558, Fisatom^®^, Fisatom, São Paulo, Brazil) under reduced pressure. The final formulation contained sesamol at a concentration of 1 mg/mL. For control purposes, placebo nanocapsules were prepared under the same conditions but without the addition of sesamol.

The hydrogel was prepared by thickening the nanocapsule suspensions. For this, the nanocapsule suspensions containing sesamol or the respective placebo formulation were placed in the glass mortar. Subsequently, guar gum (2.5% *w*/*v*) was added and dispersed using a glass pestle at room temperature until a semi-solid consistency was achieved, resulting in either Hydrogel NC Sesamol or Hydrogel NC placebo. A hydrogel containing sesamol (1 mg/g) in its free form (Hydrogel-free Sesamol) was formulated by dispersing guar gum into a sesamol aqueous solution. Additionally, the vehicle (Hydrogel vehicle) was obtained using similar procedures that involved dispersing guar gum in ultrapure water. After the homogeneous dispersion of hydrogel components, a preservative solution containing 20% methylparaben and 5% propylparaben in propylene glycol was added to all prepared hydrogels at a concentration of 2% (*w/v*). No further components were added to the hydrogels (pH corrective, antioxidants, and cross-linkers). Three batches of hydrogels were prepared per group to evaluate the parameters.

### 2.3. Fourier-Transformed Infrared (FTIR) Spectroscopy

FTIR spectroscopy was employed to explore the chemical interactions among constituents. Ten samples from each hydrogel, guar gum, sesamol, and the preservative solution underwent thorough examination using a Bruker FTIR spectrometer, specifically the Alpha-T FTIR model equipped with an Attenuated Total Reflectance (ATR) module. The spectral range covered was 4000 to 400 cm^−1^, utilizing a resolution of 4 cm^−1^ and 24 scans [26]. ATR-FTIR spectra from these sample groups were utilized to employ a chemometric model, specifically Principal Component Analysis (PCA), using MATLAB 7 and PLS-Toolbox 4 from the Eigenvector Research Group (Manson, WA, USA).

### 2.4. Chemometrics Analysis—PCA Model

Following spectrum acquisition, an unsupervised chemometrics model, PCA, was developed to differentiate sample groups, identify crucial variables, and detect potential outliers [27]. The raw data underwent pre-processing, a critical step in chemometrics that enhances data quality [28]. Once the data was pre-processed, the principal components were selected based on the eigenvalue criterion. Finally, a score plot was generated to evaluate sample discrimination and gain insights into the formulations’ chemical characteristics and potential interactions.

### 2.5. Microbiological Stability Evaluation

The microbiological evaluation of the hydrogel NC Sesamol was carried out by dispersing the formulation in phosphate buffer (pH 7.2) containing 3% (*v*/*v*) Tween 80, aiming to obtain dilutions at 10^−2^. A homogeneous hydrogel dispersion was achieved by moderate magnetic stirring for 2 h. Afterward, the dispersion was diluted to 10^−3^ in phosphate buffer (pH 7.2) containing 3% (*v*/*v*) Tween 80. Samples (1 mL) of each dilution were transferred to sterile Petri dishes (*n* = 2/dilution); then, approximately 20 mL of sterile culture medium (tryptic soy agar (TSA) for the aerobic bacteria or Sabouraud dextrose agar for the total mold and yeast counts) at 45 °C was added. After solidification, the plates were inverted and incubated. The Petri dishes were incubated at 35 ± 2.5 °C for 48 h and 25 ± 2.5 °C for 72 h to support the growth of aerobic bacteria and mold/yeast, respectively [29]. After incubation time, the number of colony-forming units (CFU) was recorded by macroscopic visualization. The requirements were a total aerobic microbial count of ≤10^2^ CFU/mL and a total combined yeast/mold count of ≤10^1^ CFU/mL [30].

### 2.6. Physicochemical Stability Evaluation

After preparation, the hydrogels were packaged in plastic containers at room temperature, and their physicochemical parameters were assessed after 15, 30, and 60 days. The average diameter and PDI of the nanocapsules present in the hydrogels, as well as the pH values and sesamol content, were measured according to the previously described methodology [23].

### 2.7. Dynamic Rheological Analysis

Rheological analysis was performed to identify the flow, elasticity, and viscosity behavior of the hydrogels using approximately 6 g of the samples. Dynamic frequency sweep and thixotropic tests were performed using a TA Instruments HR10 rheometer, which employed a cone plate geometry with a diameter of 40 mm and 2° cone angle, with a 0.3 mm gap and at 25 °C ± 2 °C, controlled by a circulating water bath. Dynamic oscillatory tests were assessed over a frequency range of 0.1 to 1000 rad/s, with a constant stress of 1 × 10^−6^ MPa. A flow ramp was used for the thixotropic performance test, where the shear rate varied from 100 to 0.1 s^−1^. The sample interval was 1.0 s/pt, and each interval was kept for 60 s.

### 2.8. Irritant Contact Dermatitis In Vivo Model

#### 2.8.1. Experimental Model

Male Swiss mice (25–30 g; 4–5 weeks of age) were produced and provided by the Central Animal Facility of the Federal University of Santa Maria. All experimental protocols were performed in accordance with national legislation (Guidelines of Brazilian Council of Animal Experimentation—CONCEA). Experimental methodologies were approved by the Institutional Animal Care and Use Committee of the Federal University of Santa Maria (CEUA #8556130623/2023).

#### 2.8.2. Experimental Design

The in vivo model of irritated contact dermatitis was designed based on preceding studies [4,31]. First, mice were randomly assigned to seven groups, with seven mice per group.

(I) Naïve group: Mice did not receive croton oil or hydrogel.
(II) Croton oil group: The mice’s right ear was sensitized by croton oil and did not receive cutaneous treatment.
(III) Hydrogel vehicle group: The mice’s right ear was sensitized by croton oil and received cutaneous applications of hydrogel vehicle.
(IV) Hydrogel NC placebo group: The mice’s right ear was sensitized by croton oil and received cutaneous applications of hydrogel NC placebo.
(V) Hydrogel-free Sesamol group: The mice’s right ear was sensitized by croton oil and received cutaneous applications of hydrogel-free sesamol.
(VI) Hydrogel NC Sesamol: The mice’s right ear was sensitized by croton oil and received cutaneous applications of hydrogel NC sesamol.
(VII) Reference drug—0.1% Dexamethasone acetate: The mice’s right ear was sensitized by croton oil and received cutaneous applications of dexamethasone acetate.

The experimental protocol was conducted over 10 days. Irritant contact dermatitis was induced by repeated cutaneous applications of croton oil (0.4 mg/ear, diluted in acetone; 20 μL/ear, applied with a micropipette) in the mice’s right ears on days 1, 3, and 5 of the experimental protocol [4,31]. Cutaneous treatments with hydrogels or dexamethasone acetate cream (0.1%; reference drug) were applied to the injured ear twice daily from day 5 until day 9 of the experimental protocol. The quantity of hydrogel used (15 mg/ear) in each treatment corresponds to a sesamol concentration of 15 µg/ear. Importantly, the mice were anesthetized with isoflurane by the inhalation route before all treatments (croton oil or hydrogels). On the last day of the experiment (day 10), the animals were euthanized with thiopental (150 mg/kg, intraperitoneal route), and the right ears were collected for histological analysis.

#### 2.8.3. Assessment of Inflammatory Markers

The mice’s right ear thickness was measured with a digital micrometer (Digimess, São Paulo, SP, Brazil) on days 5, 7, and 9 of the experimental protocol before croton oil or hydrogel treatment. An increase in ear thickness after croton oil application compared with the basal thickness (on day 1 of the experimental protocol) indicated the formation of ear edema. The results were expressed in µm. We also evaluated the inflammatory cell infiltration of the mice’s ear tissue using a histological evaluation. The number of inflammatory cells infiltrating the mice’s ears was quantified using the ImageJ software version ImageJ2 [23,32].

### 2.9. Data Presentation and Statistical Analysis

The results were expressed as mean ± standard deviation (SD) or standard error of the mean (S.E.M.). The GraphPad Prism^®^ version 8 software (Graph Pad, San Diego, CA, USA) was used to perform statistical evaluations. Initially, the normality of the data was verified using the D’Agostino & Pearson and Shapiro–Wilk normality tests. Subsequently, analysis of variance (ANOVA) followed by Tukey’s post hoc test, when applicable, were performed. Values of *p* < 0.05 were considered statistically significant.

## 3. Results

### 3.1. Fourier-Transformed Infrared (FTIR) Spectroscopy Analyzes

**Figure 1:**
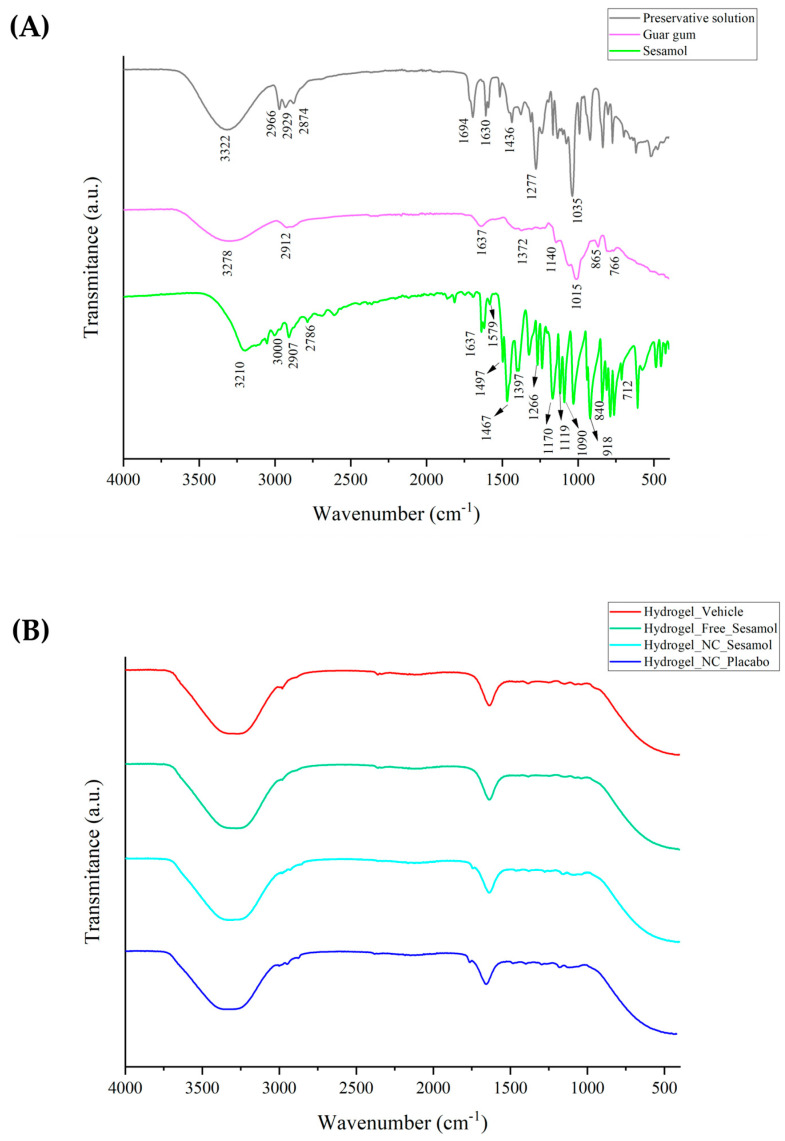
ATR-FTIR of raw materials (**A**) and hydrogels (**B**).

Figure 1 demonstrates the ATR-FTIR spectra of raw materials (A) and hydrogels (B). Sesamol spectra display observable peaks that correspond to specific functional groups. The band located at 3210 cm^−1^ is related to the phenolic O-H group. Meanwhile, the peaks at 3000 cm^−1^ and 2907 cm^−1^ are attributed to unsaturated and saturated C-H bonds, respectively. Additionally, the -CH symmetric stretching can be observed at 2786 cm^−1^, and the phenyl bonds are located at 1637 cm^−1^, 1579 cm^−1^, and 1497 cm^−1^. The peak at 1397 cm^−1^ refers to methyl symmetric bending. Finally, the peaks found at 1266 cm^−1^, 1119 cm^−1^, 1090 cm^−1^, and 918 cm^−1^ are probably due to C-O bonds. Guar gum exhibits significant peaks at specific wavelengths: 3278 cm^−1^, 2912 cm^−1^, 1140 cm^−1^, 865 cm^−1^, and 766 cm^−1^. These peaks correspond to different vibrational modes, such as O-H stretching, C-H stretching of the CH2 group, C-OH, and primary alcoholic and galactose and mannose bond linkages, respectively. For the preservative solution, peaks referring to the parabens and propylene glycol are identified. In the hydrogel formulations (Figure 1B), most of the well-defined sesamol bands are no longer detectable, particularly in the fingerprint region. This observation is consistent with the nanoencapsulation of sesamol, which likely results in the attenuation or masking of its signals due to the polymeric shell of the nanocapsules and their uniform dispersion within the matrix. Nevertheless, the broad O-H stretching band (~3300–3200 cm^−1^) remains present in all formulations, reflecting the abundance of hydrophilic functional groups from both the polymeric components and the guar gum. This spectral profile supports the successful incorporation of nanocapsules, and the formation of a structured, hydrated hydrogel network stabilized by intermolecular interactions, such as hydrogen bonding.

**Figure 2:**
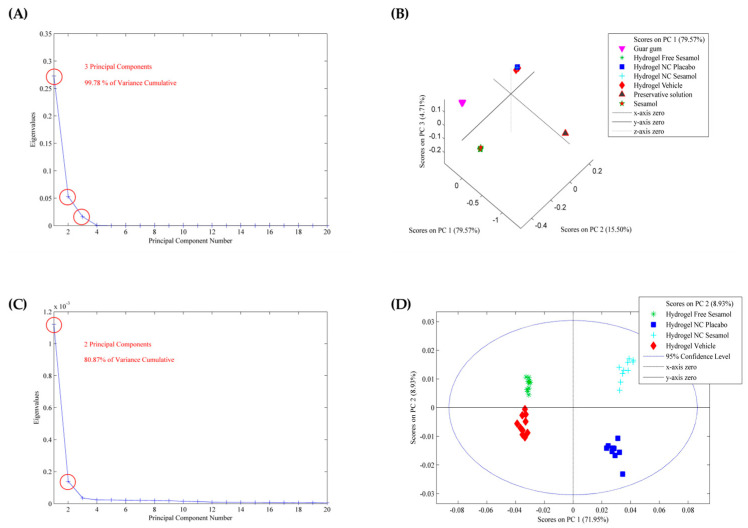
PCA model applied to the ATR-FTIR spectra of the hydrogels and raw materials. (**A**,**C**) are the eigenvalues against the number of main components, and (**B**,**D**) are the score plots.

After spectral acquisition, PCA was used to investigate the variability among hydrogel formulations based on their ATR-FTIR spectra. The absorbance data matrix was baseline-corrected and normalized to minimize noise and enhance spectral comparison. After applying smoothing and GLSW pre-processing, three components were identified as responsible for 99.78% of the cumulative variance in the data (Figure 2A). The number of principal components retained was based on the cumulative variance explained. The score graph (Figure 2B) demonstrates that the hydrogels and guar gum are in the same plane of PC2 (*y*-axis) and that the hydrogels and preservative components are in the same plane of PC1 (*x*-axis). Sesamol appears somewhat isolated from the other groups, indicating that it may be partially nanoencapsulated into the nanocapsule in the hydrogels containing sesamol-loaded nanocapsules and interacting with the gum matrix in the hydrogel containing free sesamol. With the model applied, it was impossible to visualize differences among the hydrogels. So, to better understand the formulations, PCA was performed only for the hydrogels. Smoothing and GLSW pre-processing provide two principal components that account for 80.87% of the cumulative variance (Figure 2C). It was possible to note from the score plot that both sesamol hydrogels (nano and non-nanoencapsulated) are in the same plane of PC1, suggesting a chemical similarity, probably due to the non-encapsulated portion of the sesamol (with an encapsulation efficiency of approximately 65%). The nano-based hydrogels are in the same plane as PC2 (*x*-axis), probably due to the chemical similarity of the nanocapsule suspension constituents (Figure 2D). Analysis of the loading vectors showed that the key spectral variables are mostly found in the fingerprint region (1800–900 cm^−1^), which is rich in functional group-specific vibrations and known to reflect subtle molecular interactions. These findings support the differences observed in the FTIR spectra and emphasize how formulation components affect the structure of the final hydrogel.

### 3.2. Microbiological Stability

The microbiological test demonstrated that the formulations evaluated met the pharmacopeial requirements over 60 days since the total aerobic microbial and yeasts/mold counts were <10 CFU/mL in the three batches evaluated.

### 3.3. Physicochemical Stability Evaluation

Table 1 presents the evaluation of physicochemical stability for the hydrogels. After 60 days of storage, the formulations retained the same macroscopic appearance as at their initial time. The results showed a significant increase in the average diameter of hydrogel NC Sesamol and hydrogel NC placebo after 15 and 30 days of storage. In addition, the pH values decreased significantly after 60 days for both the hydrogel NC placebo and the hydrogel vehicle. The sesamol content remained unaltered during the entire storage period.

**Figure 3:**
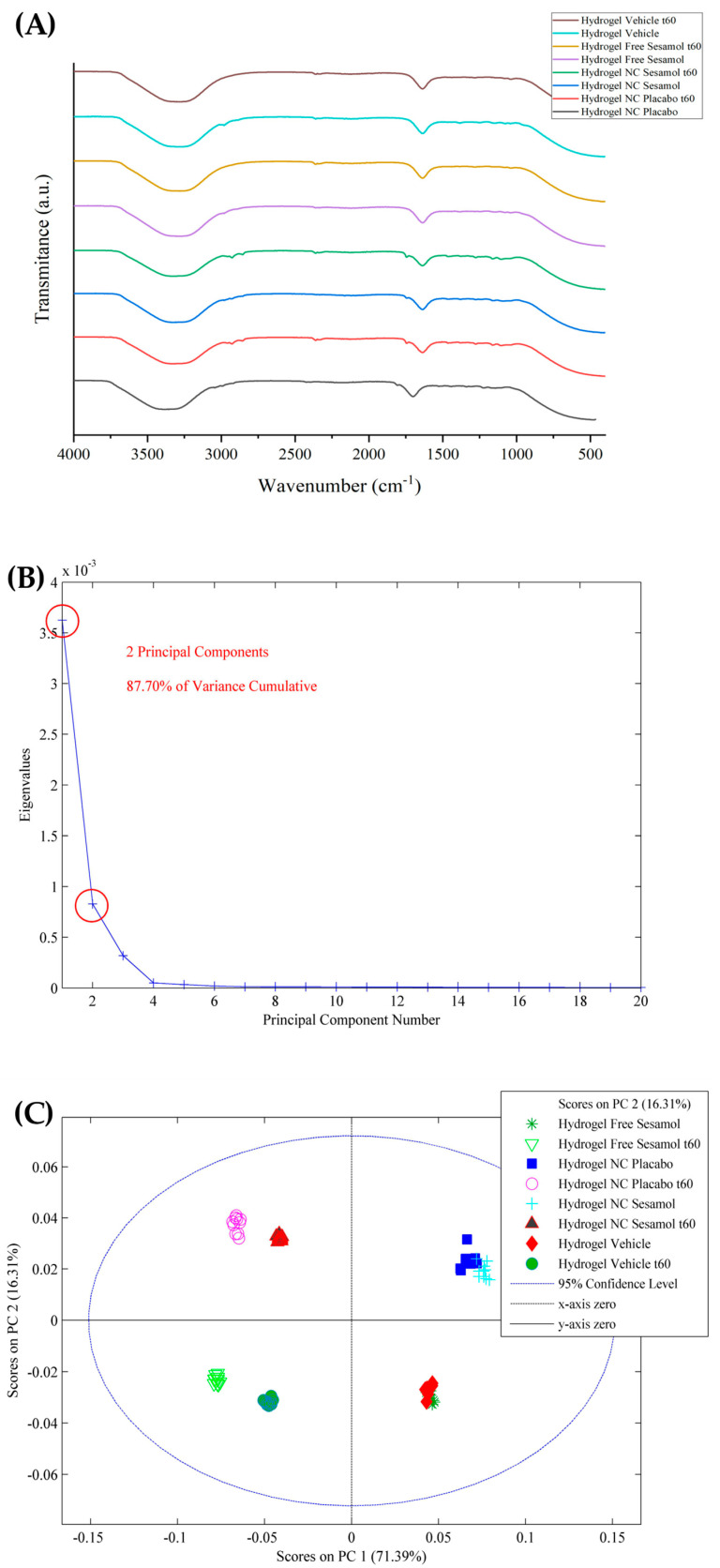
(**A**) ATR-FTIR of stability evaluation of hydrogels after 60 days of storage and PCA model applied to the spectra, wherein (**B**) are eigenvalues against the number of main components and (**C**) is the score plot.

The ATR-FTIR was also used to analyze the hydrogels after 60 days of storage. The results are depicted in Figure 3. The ATR-FTIR spectra are very similar for all formulations evaluated after preparation and 60 days of storage (Figure 3A). However, by applying the PCA model, some differences can be identified. Smoothing and GLSW pre-processing highlighted two principal components, accounting for 87.70% of the cumulative variance (Figure 3B). Analyzing the score plot (Figure 3C), the nano-based hydrogels were kept in the same plane (PC1; *y*-axis), as well as the sesamol formulations (PC2; *x*-axis), suggesting that there were no alterations. However, the evaluation plans on days 0 and 60 are the opposite for all formulations.

### 3.4. Dynamic Rheological Analysis

**Figure 4:**
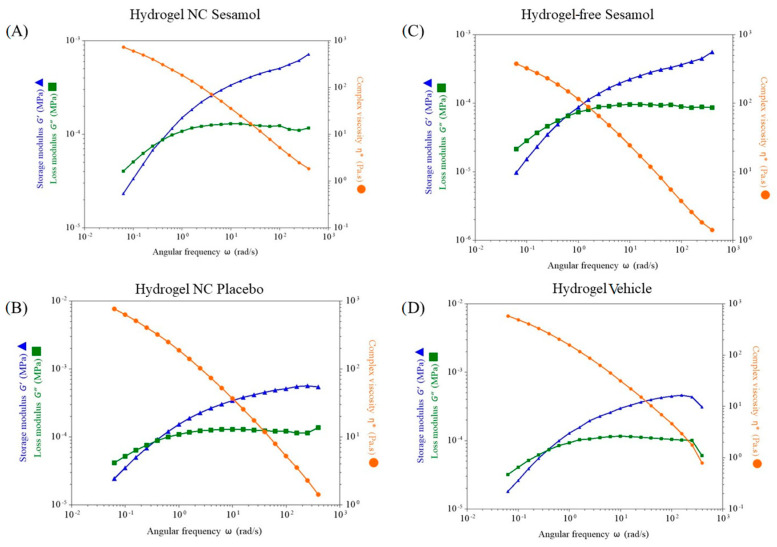
Evaluation of the hydrogels’ storage modulus (G′) and loss modulus (G″) by oscillatory rheometry. (**A**): Hydrogel NC sesamol; (**B**): Hydrogel NC placebo; (**C**): Hydrogel-free sesamol; (**D**): Hydrogel vehicle. * Complex viscosity Pa∙s.

The hydrogels were evaluated using oscillatory rheometry (Figure 4). Dynamic viscoelastic measurements of the hydrogels revealed that the storage modulus (G′) and the loss modulus (G″) were dependent on the angular frequency (rad/s). The dynamic analysis showed that the hydrogels exhibited viscous behavior at low frequencies and elastic behavior at moderate frequencies, with a crossover at approximately 0.05 rad/s. All the formulations behaved as gels, with G′ exceeding G″ at around a 0.05 angular frequency of rad/s.

**Figure 5:**
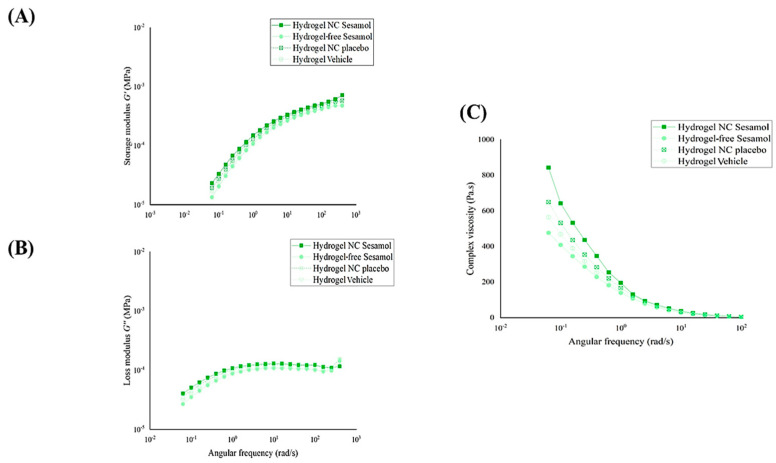
Storage modulus G′ (**A**), loss modulus G″ (**B**), and viscosity profiles of the hydrogels (**C**) were determined by oscillatory rheometry.

To illustrate the potential variance between formulations, Figure 5 displays the G′, G″, and dynamic viscosity separately. Figure 5A,B demonstrate that the G′ and G″ profiles are very similar for all formulations. In contrast, it was observed that nanocapsules had a positive impact on viscosity levels. This is demonstrated by the slightly higher complexity viscosity values in hydrogel NC sesamol, followed by hydrogel NC placebo, as shown in Figure 5C.

**Figure 6:**
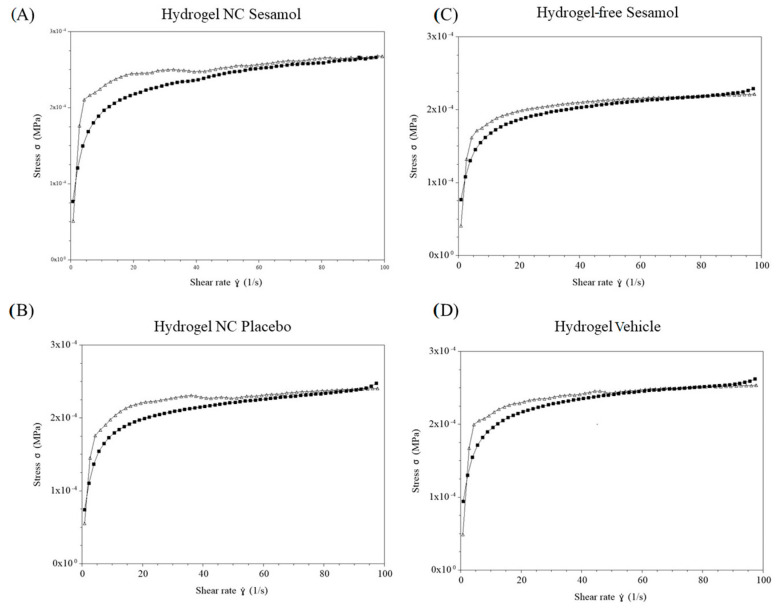
Thixotropy behavior of the hydrogels determined by oscillatory rheometry. (**A**): Hydrogel NC Sesamol; (**B**): Hydrogel NC Placebo; (**C**): Hydrogel-free Sesamol; (**D**): Hydrogel Vehicle. Black line: upward curves; gray line: downward curves.

Thixotropy was assessed through the thixotropic loop test (Figure 6). The hydrogel exhibited increasing responses with the rise in shear rate and corresponding decreases with the reduction in this rate. There is a slight difference between the upward and downward curves, suggesting that the gel’s breakdown and recovery follow similar trajectories for each formulation. It is essential to note that formulations containing the nanocapsules show a larger hysteresis area. This highlight enhanced thixotropic behavior compared with formulations without nanocapsules.

To complement the qualitative rheological analysis, a quantitative evaluation of the flow behavior was performed. Various mathematical models were initially tested to fit the shear stress versus shear rate data. Among them, the Ostwald–de Waele (Power-Law) model exhibited the best goodness of fit for all formulations, as indicated by the highest determination coefficients (r^2^ ≥ 0.939). The flow behavior indices (ղ) obtained ranged from 0.146 ± 0.003 to 0.169 ± 0.001, confirming that all hydrogels exhibited pseudoplastic (shear-thinning) behavior, characterized by decreasing viscosity with increasing shear rate. The consistency index (Ƙ), which represents the apparent viscosity at a unitary shear rate (1 s^−1^), ranged from 114.358 ± 2.561 to 134.622 ± 3.119 Pa·s. These values reflect the overall viscous strength of each formulation under flow. Together, these results, summarized in Table 2, provide robust quantitative evidence supporting the pseudoplastic nature of the hydrogels, in agreement with the trends observed in the flow curves and complex viscosity profiles (Figure 4, Figure 5 and Figure 6).

### 3.5. In Vivo Anti-Inflammatory Efficacy Assessment

**Figure 7:**
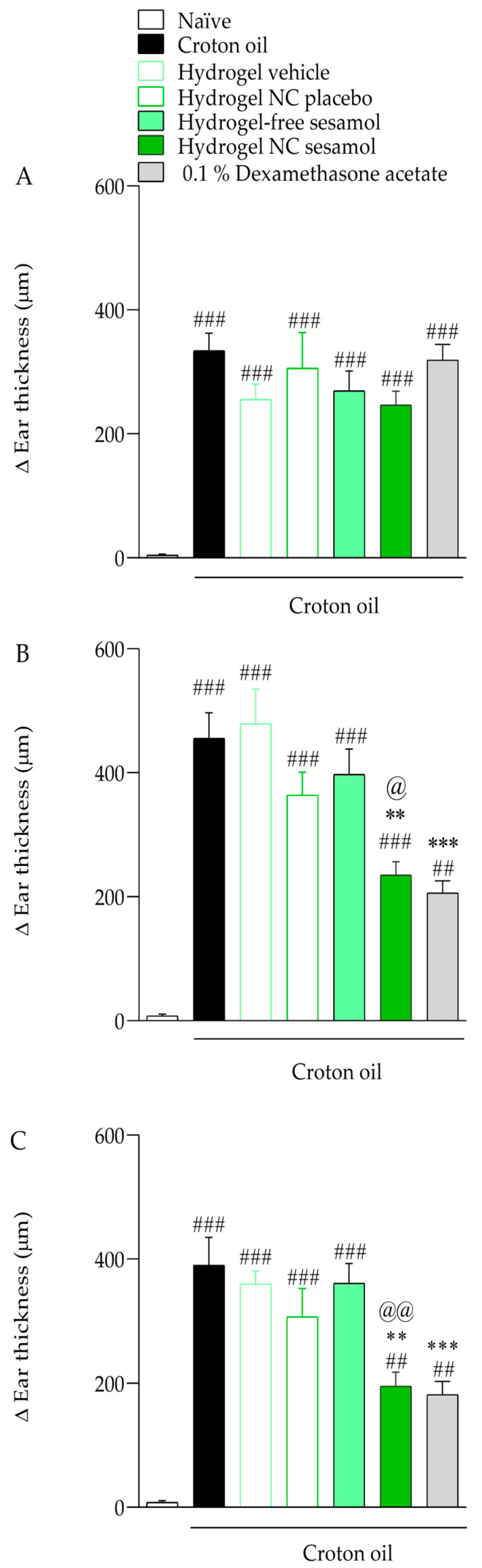
Effect of hydrogels on croton oil-induced ear edema. (**A**): Ear thickness measured on day 5 of the experimental protocol; (**B**): Ear thickness measured on day 7 of the experimental protocol; (**C**): Ear thickness measured on day 9 of the experimental protocol. Each bar represents the mean ± S.E.M. (*n* = 7). The one-way ANOVA followed by Tukey’s post hoc test was used to determine the statistical differences among the experimental groups. Sharps denote significant statistical differences compared with the naïve group (### *p* < 0.001; ## *p* < 0.01). Asterisks indicate significant statistical differences compared to the oil croton group (*** *p* < 0.001; ** *p* < 0.01). The at symbol denotes a significant statistical difference compared with the croton oil + hydrogel-free sesamol group (@@ *p* < 0.01; @ *p* < 0.05).

Figure 7 shows the antiedematogenic effects of hydrogels. Croton oil caused an increase in the mice’s ear thickness by 334.14 ± 28.17 μm, 455.30 ± 41.52 μm, and 389.90 ± 45.25 μm on days 5 (Figure 7A), 7 (Figure 7B), and 9 (Figure 7C), respectively, compared with the naïve group. On days 7 (Figure 7B) and 9 (Figure 7C) of the experimental protocol, the hydrogel NC sesamol cutaneous treatment inhibited the croton oil-induced ear thickness increase of 49.22 ± 4.82% and 50.86 ± 5.84%, while 0.1% dexamethasone acetate caused an inhibition of 55.66 ± 4.37% and 54.47 ± 5.62%, respectively. The hydrogel containing free sesamol did not significantly reduce edema formation. The inhibitory effect was calculated considering the response of the control group.

**Figure 8:**
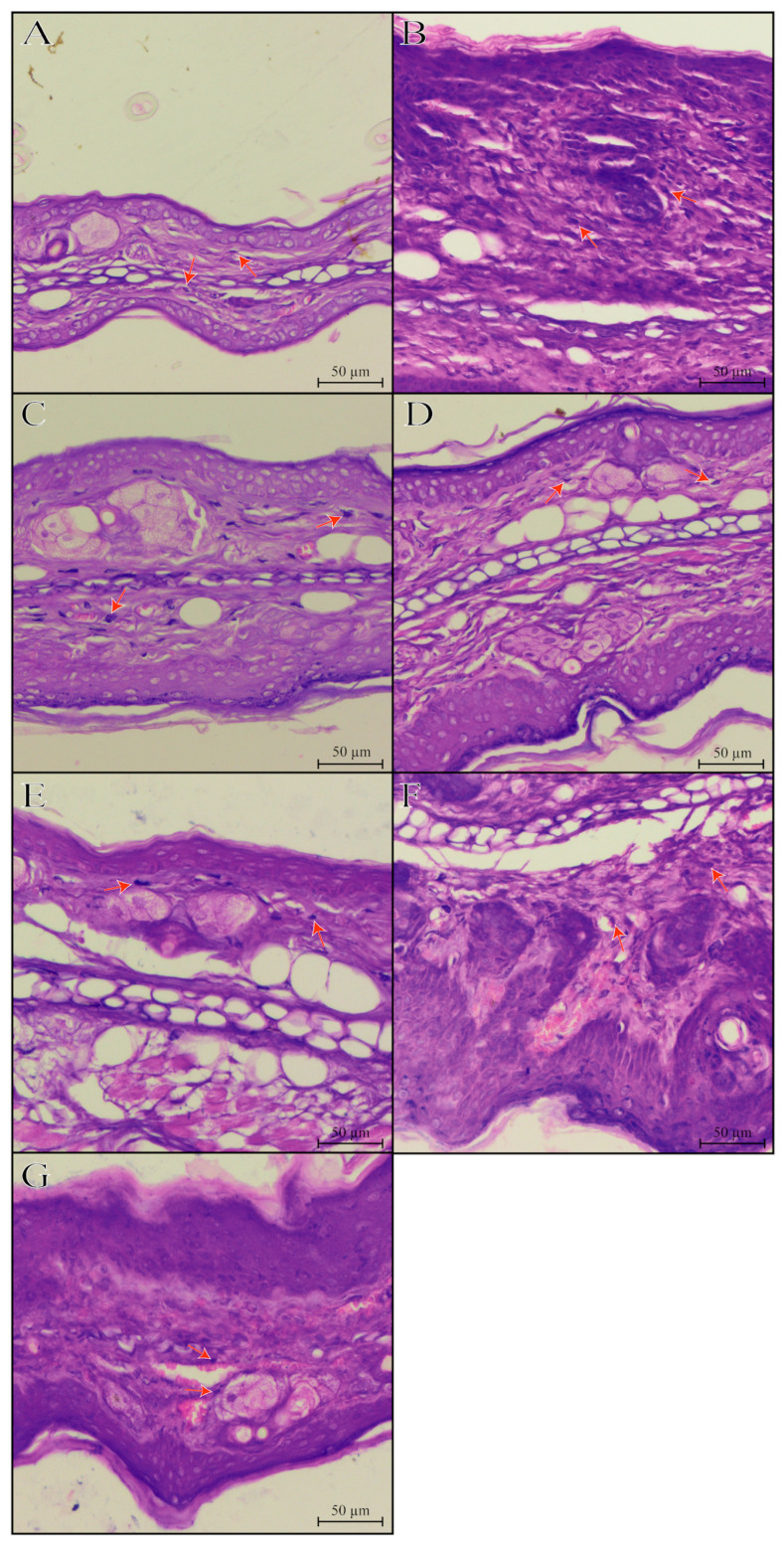
Histological vertical sections ((**A**–**G**); hematoxylin-eosin; 20× objectives) of the mice’s ear tissue. Experimental groups’ description: (**A**): representative image of the naïve group; (**B**): representative image of the croton oil group; (**C**): representative image of the croton oil + hydrogel vehicle group; (**D**): representative image of the croton oil + hydrogel NC placebo group; (**E**): representative image of the croton oil + hydrogel-free sesamol group; (**F**): representative image of the croton oil + hydrogel NC sesamol group; (**G**): representative image of the croton oil + 0.1% Dexamethasone acetate group. The orange arrows indicate the presence of inflammatory cells in the mice’s ears.

**Figure 9:**
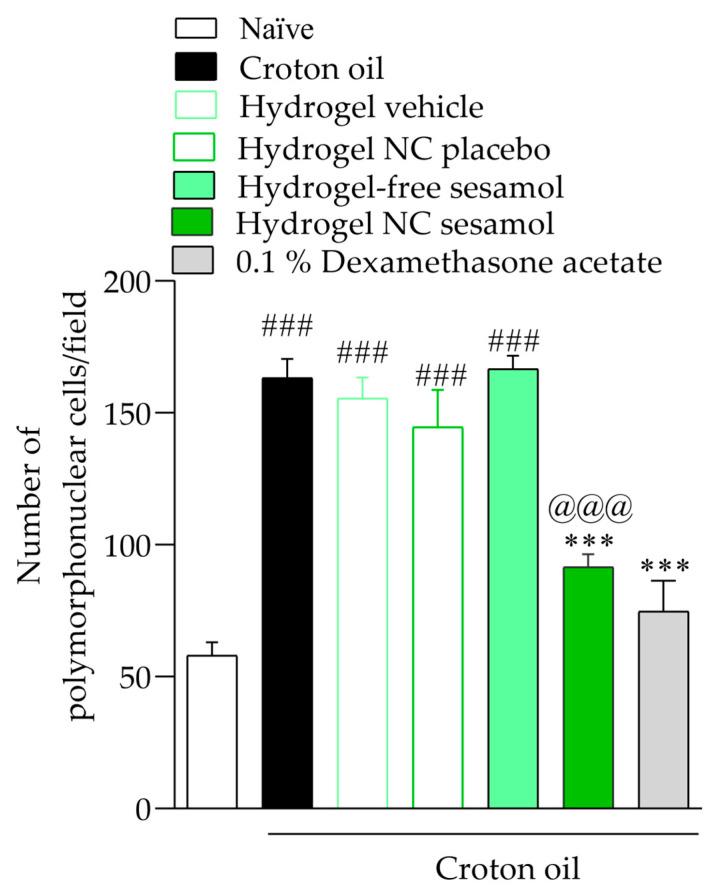
Number of polymorphonuclear cells in the mice’s ears. A one-way ANOVA followed by Tukey’s post hoc test was used to identify statistical differences among the groups. Sharps denote significant statistical differences compared with the naïve group (### *p* < 0.001). Asterisks indicate significant statistical differences compared with the oil croton group (*** *p* < 0.001). The at symbol denotes a significant statistical difference compared with the croton oil + hydrogel-free sesamol group (@@@ *p* < 0.001).

Repeated croton oil applications caused polymorphonuclear cell infiltration in the mice’s ear tissue (163 ± 9 cells per field) compared with the naïve group (58 ± 3 cells per field) (Figure 3 and Figure 4). Only hydrogel containing sesamol-loaded nanocapsules, but not free sesamol, attenuated the cell infiltration with an inhibition of 44.17 ± 1.02%. As expected, 0.1% dexamethasone acetate inhibited the polymorphonuclear cell infiltration by 70.08 ± 1.29% (Figure 8 and Figure 9).

## 4. Discussion

Dexamethasone, a corticosteroid drug, is widely used due to its potent anti-inflammatory effect. Despite this, its use in clinical practice is associated with significant adverse effects, often compromising treatment adherence [6,8]. These limitations reinforce the need to explore new therapeutic alternatives.

In this context, our previous study evaluated the therapeutic effect of nano-based hydrogel containing sesamol through a single hydrogel application in an acute model of irritant contact dermatitis [23]. Motivated by the promising results observed in the acute model, we optimized the formulation developed through physicochemical and microbiological stability studies. The results of the stability studies demonstrated that the nano-based hydrogel containing sesamol has stability, achieved by adding a preservative system. In addition, only the nano-based hydrogel containing sesamol was able to attenuate the inflammatory response in the in vivo model.

Understanding chemical reactions through analysis of ATR-FTIR spectra can be a difficult task. Given its broad spectrum of wavenumbers (4000–400 cm^−1^), each indicative of a variable, discerning subtle alterations in chemical composition can be intricate. When dealing with complex data, it is essential to utilize a chemometric model to facilitate a more straightforward interpretation of the results. The PCA tool helps reduce the data and identify prominent outliers and variables. This method is necessary for understanding the underlying patterns in the data [27,28,33].

In the present study, it was possible to note from the score plot that both hydrogels containing sesamol or sesamol-loaded nanocapsules suspension were in the same plane of PC1, suggesting a chemical similarity probably due to the non-encapsulated fraction of the sesamol. In fact, the nanocapsule suspensions had an encapsulation rate of approximately 65%, as determined in a previous study [23]. In addition, the nano-based hydrogels are positioned in the same plane as PC2 (*x*-axis), likely due to the chemical similarity of the nanocapsule’s qualitative and quantitative composition.

Microbiological and physicochemical stability assessments are important for developing new formulations [29]. A stable pharmaceutical dosage form provides greater safety and efficacy for the administered drug. In this context, we evaluated the hydrogel’s microbiological stability after adding a preservative solution. In the field of pharmaceuticals, the use of preservative agents is crucial for maintaining the microbial stability of formulations. In this context, parabens, derivatives of p-hydroxybenzoic acid, have been recognized as a potent and efficient preservative class for dosage forms for many decades, particularly due to their aqueous nature [34]. This study employed a combination of methyl and propylparaben to achieve microbiological stability over a period of 60 days.

Regarding physicochemical stability evaluations, the nano-based hydrogels exhibited a slight increase in average diameter and polydispersity index (PDI) values, likely due to particle aggregation and interactions between guar gum chains. Corroborating this, previous studies have reported similar physicochemical alterations in nano-based hydrogels developed by thickening nanoformulations with natural gums [35,36]. In addition, the ATR-FTIR stability evaluation of the hydrogels after 60 days of storage suggested no alterations, corroborating the physicochemical evaluations, where the sesamol content remained unchanged. Despite this, the evaluation planes for days 0 and 60 are the opposite for all formulations. It could be due to a slight decrease in pH values, which may have influenced the chemical characteristics of the components.

The analysis of rheological behavior provides an estimate of the flow and deformation behavior of the hydrogels. The results allow classification into two types of flow behavior: Newtonian and non-Newtonian. Newtonian fluids have a constant consistency regardless of the force applied. On the other hand, in non-Newtonian fluids, the consistency is changed proportionally to the applied force. This change profile can be classified as plastic, pseudoplastic, or dilatant behavior [37]. A proportional decrease in the consistency of the formulation concerning the applied force characterizes pseudoplastic behavior. This profile is suitable for a formulation designed for cutaneous application, as it facilitates application to the injured site [4]. In the present study, rheological analysis revealed that increasing the shear rate resulted in a decrease in the viscosity of the hydrogels. Therefore, both hydrogels exhibited non-Newtonian flow behavior.

Frequency sweep experiments are a practical method for studying the rheology of hydrogels. These experiments provide valuable information regarding the material’s crosslinking behavior and the presence of a reversible network. Our observations suggest that at a certain critical point, as the stress is increased, the G′-G″ crossover point may occur, leading to a gel–sol transformation. This behavior indicates a reversible crosslinking, which was verified by the results of the thixotropic determination. Although both formulations had similar G′ and G″ values, the nano-based hydrogels were more viscous and had higher recovery capacity than the non-nano-based hydrogels, as shown by thixotropic evaluations.

After optimizing the developed hydrogel, we investigated the therapeutic effect of the formulations in an in vivo dermatitis model. In this context, our current findings demonstrate that the administration of croton oil induced an inflammatory process according to the parameters evaluated. The 12-O-tetradecanoylphorbol-13-acetate (TPA), the main constituent of croton oil, mediates dermatitis development by increased vascular permeability, activation of inflammatory pathways, and proliferation of epidermal keratinocytes [4,23,32,38,39,40,41]. In contrast, the cutaneous application of the nano-based hydrogel containing sesamol mitigated the inflammatory manifestations.

Previous studies have investigated the anti-inflammatory mechanism of sesamol in both in vitro and in vivo models [14]. In this context, a preceding study by Chu and coworkers [42] evaluated molecular and biochemical parameters to elucidate the anti-inflammatory action of sesamol in lipopolysaccharide-induced inflammatory responses in RAW 264.7 macrophage cells.

Sesamol treatment of macrophage cells reduced the cyclooxygenase-2 (COX-2)-mediated synthesis of prostaglandins and reactive oxygen species production after lipopolysaccharide stimulation in macrophage cells. Moreover, the mRNA expression and levels of nitric oxide synthase (iNOS) and COX-2 in macrophages were reduced by sesamol pretreatment. Similarly, the generation of tumor necrosis factor-α (TNF-α) as well as the pro-inflammatory interleukin-1β (IL-1β) and IL-6 levels were all attenuated by sesamol treatment.

In this sense, we recognize the need for additional inflammatory biochemical and molecular markers, as well as the evaluation of pharmacological mechanisms, in this study. However, our study was designed to optimize the hydrogel developed through stability evaluations and evaluate the therapeutic effect of hydrogels through repeated cutaneous applications.

In this context, our results demonstrate that only the hydrogel containing sesamol nanocapsules has an effect on inflammatory manifestations. In this sense, the results corroborate the findings of our previous study, in which we evaluated the effectiveness of the hydrogel in an acute irritant contact dermatitis model [23]. Considering the physicochemical properties of sesamol, the results of the in vivo model, and the evaluation of sesamol’s permeation rate in human skin, we hypothesize that the hydrogel containing sesamol in its free form presents rapid permeation through the skin, thereby limiting its residence in the skin layers [23]. On the other hand, the hydrogel containing sesamol in nanoencapsulated form showed a more sustained release profile and, consequently, a lower permeation rate of this compound.

Corroborating the pharmacological effects observed in our previous study, the free-form hydrogel containing sesamol showed no significant effect despite repeated administration. Therefore, these results reinforce our hypothesis that the cutaneous administration of this compound in its free form results in its rapid skin permeation, thereby limiting its pharmacological action on injured skin.

Finally, sesamol demonstrated efficacy without visible signs of irritation, suggesting good local tolerability. Moreover, the nanoencapsulation of sesamol decreases the rate of permeation through skin layers, enhancing safety for repeated applications. However, we acknowledge that long-term safety data, particularly regarding cumulative exposure, immunological sensitization, or potential effects in skin barrier recovery, are still limited. Future studies will be necessary to assess chronic exposure protocols, investigate possible impacts on skin homeostasis and regeneration, and confirm the absence of local or systemic toxicity in prolonged use. These aspects are essential for advancing sesamol-based formulations before clinical studies.

## 5. Conclusions

In conclusion, adding the preservative solution to the hydrogels provided microbiological stability to the formulation. Consequently, this allowed us to advance knowledge about the physicochemical parameters of the hydrogels and investigate the possible interactions between the raw materials in more detail. Assessments of the physicochemical parameters evaluated in the present study indicate that the nano-based hydrogel exhibits satisfactory stability, with no significant changes under the conditions assessed. In addition, nano-based hydrogel containing sesamol attenuated the inflammatory manifestations induced by the croton oil. These results reinforce the benefits of sesamol nanoencapsulation, with the aim of its cutaneous application in inflammatory conditions that affect human skin.

## Figures and Tables

**Table 1:** Physicochemical stability of hydrogels.

	Parameters			
	AD ^a^(nm)	PDI ^b^	pH	Sesamol Content (%—1 mg/g)
	Initial time			
Hydrogel NC Sesamol	131 ± 1	0.11 ± 0.02	6.40 ± 0.13	97.9 ± 1.5
Hydrogel NC placebo	133 ± 3	0.12 ± 0.01	6.38 ± 0.05	- ^c^
Hydrogel vehicle	-	-	6.32 ± 0.11	-
Hydrogel-free Sesamol	-	-	6.47 ± 0.23	97.4 ± 0.3
	After 15 days			
Hydrogel NC Sesamol	148 ± 10	0.15 ± 0.03	6.01 ± 0.10	97.4 ± 0.6
Hydrogel NC placebo	145 ± 3 *	0.15 ± 0.04	5.90 ± 0.06	-
Hydrogel vehicle	-	-	6.32 ± 0.11	-
Hydrogel-free Sesamol	-	-	6.47 ± 0.23	96.1 ± 2.3
	After 30 days			
Hydrogel NC Sesamol	175 ± 9 *	0.20 ± 0.12	6.06 ± 0.03	96.0 ± 0.8
Hydrogel NC placebo	161 ± 2 *	0.19 ± 0.01	5.97 ± 0.04	-
Hydrogel vehicle	-	-	6.32 ± 0.11	-
Hydrogel-free Sesamol	-	-	6.47 ± 0.23	95.9 ± 1.6
	After 60 days			
Hydrogel NC Sesamol	200 ± 19	0.24 ± 0.11	5.83 ± 0.05	96.6 ± 2.5
Hydrogel NC placebo	208 ± 38	0.30 ± 0.10	5.86 ± 0.06 *	-
Hydrogel vehicle	-	-	5.83 ± 0.09 *	-
Hydrogel-free Sesamol	-	-	5.71 ± 0.17	96.4 ± 1.5

The values are expressed as mean ± SD of three batches per hydrogel. The data were analyzed using a one-way ANOVA of repeated measures. * *p* < 0.05 denotes the significant difference compared with its initial time. Abbreviations: AD ^a^: average diameter; PDI ^b^: Polydispersity index; - ^c^: not applicable.

**Table 2:** Flow behavior index (*n*), consistency index (Ƙ), and corresponding r^2^ values of the hydrogels.

Parameters	Hydrogel NC Sesamol	Hydrogel NC Placebo	Hydrogel Vehicle	Hydrogel-Free Sesamol
Mathematical model	Ostwald–de Waele	Ostwald–de Waele	Ostwald–de Waele	Ostwald–de Waele
r^2^	0.942 ± 0.005	0.939 ± 0.030	0.940 ± 0.006	0.944 ± 0.004
Consistency index (Ƙ)—Pa∙s	118.599 ± 6.984	114.358 ± 2.561	134.622 ± 3.119	119.677 ± 7.514
Flow index (ղ)	0.169 ± 0.001	0.163 ± 0.001	0.146 ± 0.003	0.151 ± 0.007

The values are expressed as mean ± SD of three batches per hydrogel.

## Data Availability

Data will be made available on request.

## Abbreviations

The following abbreviations are used in this manuscript:

NC	Nanocapsules
FTIR	Fourier-transformed infrared
TSA	Tryptic soy agar
PCA	Principal Component Analysis
